# Supplementary material for: Thermal Inactivation of Different Capripox Virus Isolates
Source: Microorganisms. 2020 Dec 21;8(12):2053. doi: 10.3390/microorganisms8122053 (PMC7767500; doi:10.3390/microorganisms8122053)
Supplement: Supplementary file 1 [file microorganisms-08-02053-s001.pdf]

Supplemental Table S1: Viral genome loads (Cq-values) of different capripox virus strains in different media after thermal treatment.

LSDV vaccine strain "Neethling"				LSDV field strain "Macedonia2016"				GTPV field strain "V/103"				SPPV vaccine strain "V/104"			
Cq-value				Cq-value				Cq-value				Cq-value			
**#**	**PBS**	**ZB**	**FCS**	**#**	**PBS**	**ZB**	**FCS**	**#**	**PBS**	**ZB**	**FCS**	**#**	**PBS**	**ZB**	**FCS**
**120 min 4°C**				**120 min 4°C**				**120 min 4°C**				**120 min 4°C**			
Start	20.5	20.5	20.5	Start	21.0	20.7	20.4	Start	23.1	23.0	22.9	Start	23.7	23.2	23.2
#1	20.2	20.3	19.7	#1	20.0	19.7	18.2	#1	22.1	21.1	21.0	#1	25.0	24.8	23.1
#2	22.4	21.7	21.4	#2	21.1	21.8	20.7	#2	22.4	22.7	22.0	#2	25.9	25.3	24.3
#3	20.1	20.1	20.1	#3	19.5	19.5	19.3	#3	22.5	22.8	22.9	#3	26.0	25.8	24.4
**5 min 56°C**				**5 min 56°C**				**5 min 56°C**				**5 min 56°C**			
Start	20.5	20.1	20.2	Start	20.9	20.4	20.2	Start	23.1	22.5	22.6	Start	24.0	23.6	23.3
#1	20.3	20.6	20.2	#1	20.2	20.8	19.1	#1	22.4	22.1	21.2	#1	23.1	23.3	21.8
#2	22.4	23.1	22.0	#2	20.5	21.3	21.9	#2	23.2	22.7	22.5	#2	26.3	26.1	23.5
#3	20.3	21.0	20.0	#3	20.6	22.0	22.1	#3	23.0	22.7	22.1	#3	26.4	25.1	24.3
**10 min 56°C**				**10 min 56°C**				**10 min 56°C**				**10 min 56°C**			
Start	20.5	20.5	20.9	Start	20.7	20.3	21.1	Start	23.1	22.7	23.4	Start	23.8	23.4	23.5
#1	21.3	22.1	23.5	#1	24.1	25.7	22.7	#1	23.8	22.6	21.0	#1	26.1	26.2	22.5
#2	21.5	20.5	22.1	#2	22.5	22.3	22.3	#2	22.7	23.0	24.3	#2	26.3	25.5	24.2
#3	20.9	21.8	21.5	#3	22.7	22.5	20.1	#3	22.6	22.3	22.4	#3	22.4	22.4	23.7
**30 min 56°C**				**30 min 56°C**				**30 min 56°C**				**30 min 56°C**			
Start	21.1	19.4	20.2	Start	20.6	20.7	22.1	Start	23.2	23.3	23.7	Start	23.5	23.8	24.2
#1	27.4	27.6	28.3	#1	28.6	27.7	29.2	#1	28.5	27.3	28.7	#1	27.2	27.0	27.6
#2	31.1	30.8	31.1	#2	31.4	31.3	31.4	#2	30.8	29.6	30.0	#2	29.4	29.1	29.1
#3	33.6	33.2	35.1	#3	34.8	33.8	34.7	#3	31.1	32.2	31.9	#3	31.3	31.4	31.1
**60 min 56°C**				**60 min 56°C**				**60 min 56°C**				**60 min 56°C**			
Start	20.3	20.2	21.9	Start	20.7	20.6	22.4	Start	23.5	23.3	24.9	Start	23.5	23.4	24.1
#1	28.0	28.0	27.6	#1	28.5	28.3	29.5	#1	28.6	27.9	28.5	#1	27.4	27.2	27.8
#2	31.2	30.5	30.6	#2	31.9	31.3	31.4	#2	31.1	30.0	29.5	#2	28.9	28.1	28.5
#3	34.4	33.0	33.7	#3	35.5	33.7	35.0	#3	34.1	32.9	32.0	#3	31.3	31.8	31.6
**120 min 56°C**				**120 min 56°C**				**120 min 56°C**				**120 min 56°C**			
Start	20.8	20.5	21.5	Start	21.1	21.2	23.0	Start	23.2	23.4	25.4	Start	23.6	23.5	24.6
#1	28.0	28.0	28.4	#1	28.7	28.3	29.4	#1	29.1	28.1	28.6	#1	27.2	27.3	27.7
#2	31.0	30.3	30.6	#2	31.8	30.2	31.6	#2	30.2	30.0	29.7	#2	28.8	28.1	28.5
#3	34.1	33.1	33.3	#3	35.3	33.7	35.8	#3	33.7	31.6	32.0	#3	31.3	31.4	31.4
**5 min 60°C**				**5 min 60°C**				**5 min 60°C**				**5 min 60°C**			
Start	20.4	19.9	20.3	Start	20.8	20.4	20.5	Start	22.0	22.9	23.3	Start	23.5	23.4	23.5
#1	27.4	27.4	28.3	#1	28.3	28.8	29.4	#1	28.1	28.0	28.4	#1	27.5	27.1	27.5
#2	26.2	30.4	28.0	#2	31.4	31.0	28.9	#2	30.6	29.7	26.1	#2	29.3	28.9	28.3
#3	19.3	22.1	20.1	#3	25.1	34.3	17.8	#3	33.6	32.0	22.7	#3	31.7	31.2	31.5
**10 min 60°C**				**10 min 60°C**				**10 min 60°C**				**10 min 60°C**			
Start	20.5	19.9	20.9	Start	20.6	20.2	21.3	Start	23.1	22.6	23.5	Start	23.4	23.2	23.6
#1	26.9	27.5	28.3	#1	28.7	28.5	30.2	#1	28.3	27.5	28.8	#1	27.0	27.1	27.8
#2	30.3	30.8	30.1	#2	31.8	31.0	31.8	#2	31.1	29.2	29.4	#2	28.3	27.8	28.4
#3	34.6	33.9	33.5	#3	35.4	33.6	34.1	#3	33.2	32.3	32.1	#3	31.6	31.4	31.7

displays cell culture passage

Supplemental Table S2: Viral genome loads (Cq-values) of the used PCPV isolate in different media after thermal treatment.

Parapox bovis 2 (PCPV)							
Cq-value				Cq-value			
#	PBS	ZB	FCS	#	PBS	ZB	FCS
120 min 4°C							
Start	25.2	25.2	25.0				
#1	19.9	20.4	20.3				
#2	20.7	21.9	21.8				
#3	21.7	21.8	21.7				
5 min 56°C				5 min 60°C			
Start	25.3	25.1	24.1	Start	25.3	24.6	24.3
#1	19.7	20.4	20.4	#1	20.6	21.5	19.9
#2	20.9	21.1	20.3	#2	21.4	21.2	21.3
#3	21.5	21.3	21.3	#3	21.5	20.8	20.8
10 min 56°C				10 min 60°C			
Start	25.0	24.5	24.5	Start	25.2	24.8	24.5
#1	19.5	19.9	19.6	#1	24.1	24.9	24.7
#2	20.8	20.6	21.6	#2	21.1	21.8	21.6
#3	21.2	21.1	21.6	#3	20.7	20.9	21.5
30 min 56°C				30 min 60°C			
Start	25.1	24.6	25.2	Start	24.7	23.7	24.7
#1	23.0	23.9	21.6	#1	29.2	30.1	29.2
#2	21.2	21.5	21.0	#2	32.2	32.3	33.0
#3	21.9	21.4	21.3	#3	35.4	34.6	35.5
60 min 56°C				60 min 60°C			
Start	25.3	24.6	25.4	Start	24.6	24.1	25.4
#1	28.0	27.6	25.8	#1	29.1	29.7	29.0
#2	19.9	19.2	20.3	#2	32.7	32.8	32.9
#3	21.4	21.1	20.8	#3	37.5	34.8	35.1
120 min 56°C				120 min 60°C			
Start	25.4	24.9	26.4	Start	24.6	24.2	25.7
#1	30.5	30.9	31.3	#1	29.2	28.4	29.4
#2	32.6	33.4	26.0	#2	32.2	32.3	32.8
#3	35.7	36.8	20.5	#3	34.9	34.1	no Cq
180 min 56°C				180 min 60°C			
Start	24.9	24.5	26.3	Start	24.6	24.5	26.1
#1	29.4	29.4	30.3	#1	29.1	29.5	29.3
#2	32.3	31.7	32.3	#2	32.3	32.1	32.8
#3	35.8	35.2	35.1	#3	35.0	34.6	36.3
240 min 56°C				240 min 60°C			
Start	24.6	25.1	26.5	Start	24.4	24.7	26.1
#1	29.2	29.9	30.0	#1	29.1	30.0	29.6
#2	32.4	31.3	32.7	#2	32.4	32.5	33.1
#3	37.1	34.7	36.3	#3	36.2	37.0	36.3

displays cell culture passage

Supplemental Table S3: Uninfected cell culture served as negative control during passaging of heat-treated virus suspensions. Both the pan-capripox and the Parapox-B2L real-time qPCR assays were used to detect possible contaminations during passaging or molecular analyses of samples. Internal control DNA (EGFP) served as control for successful DNA extraction.

Cell culture control			
**Cq value**			
**pan Capri-p32-Mix1-Taq-FAM**	**#1**	**#2**	**#3**
MDBK control 1	no Cq	no Cq	no Cq
MDBK control 2	no Cq	no Cq	no Cq
MDBK control 3	no Cq	no Cq	no Cq
**EGFP-Mix1(5)-HEX (capripox)**	**#1**	**#2**	**#3**
MDBK control 1	27.9	26.5	27.9
MDBK control 2	28.0	26.6	27.8
MDBK control 3	27.8	27.0	27.7
**Parapox-B2L-Mix-MGB-FAM**	**#1**	**#2**	**#3**
MDBK control 1	no Cq	no Cq	no Cq
MDBK control 2	no Cq	no Cq	no Cq
MDBK control 3	no Cq	no Cq	no Cq
**EGFP-Mix1(5)-HEX (parapox)**	**#1**	**#2**	**#3**
MDBK control 1	26.4	25.8	26.3
MDBK control 2	26.1	24.9	26.2
MDBK control 3	26.5	25.2	26.3
**pan Capri-p32-Mix1-Taq-FAM**	**#1**	**#2**	**#3**
SFT-R control 1	no Cq	no Cq	no Cq
SFT-R control 2	no Cq	no Cq	no Cq
SFT-R control 3	no Cq	no Cq	no Cq
**EGFP-Mix1(5)-HEX (capripox)**	**#1**	**#2**	**#3**
SFT-R control 1	27.7	27.1	27.6
SFT-R control 2	28.0	27.1	27.7
SFT-R control 3	28.0	27.3	27.9
**Parapox-B2L-Mix-MGB-FAM**	**#1**	**#2**	**#3**
SFT-R control 1	no Cq	no Cq	no Cq
SFT-R control 2	no Cq	no Cq	no Cq
SFT-R control 3	no Cq	no Cq	no Cq
**EGFP-Mix1(5)-HEX (parapox)**	**#1**	**#2**	**#3**
SFT-R control 1	26.5	25.7	26.1
SFT-R control 2	26.3	26.2	26.1
SFT-R control 3	26.5	25.8	26.1

displays cell culture passage
